# From Selye’s and Szabo’s Cysteamine-Duodenal Ulcer in Rats to Dopamine in the Stomach: Therapy Significance and Possibilities

**DOI:** 10.3390/ph16121699

**Published:** 2023-12-07

**Authors:** Predrag Sikiric, Alenka Boban Blagaic, Ivan Krezic, Helena Zizek, Luka Kalogjera, Ivan Maria Smoday, Vlasta Vukovic, Katarina Oroz, Helen Marie Chiddenton, Sara Buric, Marko Antunovic, Slaven Gojkovic, Sanja Strbe, Milena Skocic, Suncana Sikiric, Marija Milavic, Lidija Beketic Oreskovic, Antonio Kokot, Antun Koprivanac, Ivan Dobric, Marko Sever, Mario Staresinic, Lovorka Batelja Vuletic, Anita Skrtic, Sven Seiwerth

**Affiliations:** 1 Department of Pharmacology, School of Medicine, University of Zagreb, 10000 Zagreb, Croatia; abblagaic@mef.hr (A.B.B.); ivankrezic94@gmail.com (I.K.); zizekhelena@gmail.com (H.Z.); lkalogjera9@gmail.com (L.K.); ivansmoday1@gmail.com (I.M.S.); vukovic.vlasta1@gmail.com (V.V.); oroz.kat@hotmail.com (K.O.); hm.chiddenton@gmail.com (H.M.C.); saraburic3d@hotmail.com (S.B.); slaven.gojkovic.007@gmail.com (S.G.); strbes@gmail.com (S.S.); milena.skocic@gmail.com (M.S.); lidijabeketicoreskovic@gmail.com (L.B.O.); akoprivanac@gmail.com (A.K.); 2 Department of Pathology, School of Medicine, University of Zagreb, 10000 Zagreb, Croatia; suncanasikiric@gmail.com (S.S.); marija.milavic@mef.hr (M.M.); lbatelja@mef.hr (L.B.V.); sven.seiwerth@mef.hr (S.S.); 3 Department of Anatomy and Neuroscience, Faculty of Medicine, Josip Juraj Strossmayer University of Osijek, 31000 Osijek, Croatia; antonio.kokot@mefos.hr; 4 Department of Surgery, School of Medicine, University of Zagreb, 10000 Zagreb, Croatia; ivandobricmd@gmail.com (I.D.); dr.sever.marko@gmail.com (M.S.)

**Keywords:** cysteamine, dopamine agonists, dopamine antagonists, gastroprotection, peptides, amylin, cholecystokin, leptin, BPC 157

## Abstract

We reviewed gastric ulcer healing by dopamine considering several distinctive duodenal key points. Selye and Szabo describe the cysteamine-induced duodenal ulcer in rats as a duodenal stress ulcer in patients. Szabo’s cysteamine duodenal ulcer as the dopamine duodenal healing and cysteamine as a dopamine antagonist signifies the dopamine agonists anti-ulcer effect and dopamine antagonists ulcerogenic effect. From these viewpoints, we focused on dopamine and gastric ulcer healing. We mentioned antecedent studies on the dopamine presence in the stomach and gastric juice. Then we reviewed, in the timeline, therapy significance arising from the anti-ulcer potency of the various dopamine agonists, which is highly prevailing over the quite persistent beneficial evidence arising from the various dopamine antagonists. Meanwhile, the beneficial effects of several peptides (i.e., amylin, cholecystokinin, leptin, and stable gastric pentadecapeptide BPC 157, suggested as an acting mediator of the dopamine brain-gut axis) were included in the dopamine gastric ulcer story. We attempt to resolve dopamine agonists/antagonists issue with the dopamine significance in the stress (cysteamine as a prototype of the duodenal stress ulcer), and cytoprotection (cysteamine in small dose as a prototype of the cytoprotective agents; cysteamine duodenal ulcer in gastrectomized rats). Thereby, along with dopamine agonists’ beneficial effects, in special circumstances, dopamine antagonists having their own ulcerogenic effect may act as “mild stress (or)” or “small irritant” counteracting subsequent strong alcohol or stress procedure-induced severe lesions in this particular tissue. Finally, in the conclusion, as a new improvement in further therapy, we emphasized the advantages of the dopamine agents’ application in lower gastrointestinal tract therapy.

## 1. Introductory Remarks by Selye and Szabo

This review focused on the dopamine in the stomach, therapy significance, and possibilities, as a still unrealized undertaking.

The story initiated the description of the cysteamine-induced duodenal ulcer in rats by Selye and Szabo [1] closely corresponding to the duodenal ulcer in patients [1]. The message may indeed be over the questionable complexity and reliability of previous methods for duodenal ulcer induction in rats. Yet, before, several methods were used to induce duodenal ulcers. There was Robert’s continuous subcutaneous infusion of various secretagogues such as histamine, carbachol, and pentagastrin, simultaneous application of several of these agents during fasting to obtain a high incidence, or the constant perfusion of the stomach and duodenum with acid after the introduction of a permanent catheter through a skin incision in fasted and immobilized rats [2,3,4].

Of note, Selye and Szabo’s description of the cysteamine duodenal ulcer in rats [1] starts with the quotation of Selye’s original stress introductory description in Nature [5], stress in itself, or in combination with various sensitizing factors as the main ulcerogenic means [1]. In their “stress view”, the cysteamine duodenal ulcers in rats will get over the problem arising from the multiple gastric erosions as the most characteristic rat gastrointestinal manifestations of exposure to stress, and would closely mimic human “stress ulcers”, which are frequently localized in the duodenum [1]. They considered various agents in duodenal ulcer potency, and thereby, they emphasized also “some relation to nonspecific stress” since the cysteamine was the most potent agent of the many other agents (acetanilide, allylchloride, acetaminophen, 4,4-diamino-diphenylmethane, propionitrile, and 3,4-toluene diamine) capable of inducing such lesions [1]. Yet, at that time, no mention was made of any influence of dopamine or gastric acid secretion [1]. With such particular “stress” notation to the duodenal lesions [1], initiation goes along with the emergence of the histamine, and H2 receptors blockers resolution of the peptic ulcer therapy [6]. Furthermore, Szabo’s subsequent cysteamine report in the Lancet revealed the dopamine and gastric acid hypersecretion background and it became a seminal dopamine paper [7].

It appeared almost simultaneously with Robert’s introduction of the concept of cytoprotection by prostaglandins [8]. Essentially, Robert claimed cytoprotection as a beneficial effect, which is not gastric acid-dependent, stomach cell protection against various nocuous agents applied intragastrically (i.e., absolute ethanol used as a strong irritant), and severe gastric lesions [8]. Later, Robert introduced an additional concept, adaptive cytoprotection, providing that the initial application of the small irritant (i.e., 15% alcohol) and its mild ulcerogenic effect protect against subsequent strong irritant (and severe ulcerogenic effect) [9]. Interestingly, the cytoprotection was also coined to the cysteamine [10]. While cysteamine acts in the high dose range as a dopamine antagonist and duodenal ulcerogen [2], given in lower doses, it has a positive effect on the gastric mucosa challenged with strong alcohol known as cytoprotection [10]. This was, however, attributed to its sulfhydryl capability [10] while the term cytoprotection was used in dopamine agonist stress studies [11,12].

Importantly, the Selye and Szabo “stress study” [1], subsequently presented as the dopamine story [7], influences both concepts, “no acid, no ulcer” (i.e., cysteamine as a “classic” gastric hypersecretion-induced duodenal ulcer model [7]), and “gastric acid-nondependent protection” [10], and vice versa. These concepts may influence the dopamine story. However, there is an additional huge conceptual distinction with the dopamine concept. The advantage accompanies the contention of Parkinson’s disease/schizophrenia/ulcers. Parkinson’s disease patients have an increased incidence of ulcers. Schizophrenia patients have a lesser incidence of ulcers. Thereby, dopamine agonists may be suited to antiulcer agents [7,13]. In addition, cysteamine, being a dopamine antagonist and ubiquitous industrial pollution, explains the increased incidence of peptic ulcer disease with the increased industrialization [13]. Again, the emphasis is on the dopamine agonist application [13]. Furthermore, an important etiologic and pathogenetic point of view accompanies the structure-activity studies [13]. Cysteamine, propionitrile, and their derivatives, as well as with analogs of toluene, revealed numerous alkyl and aryl duodenal ulcerogens, much like the dopaminergic neurotoxin 1-methyl-4-phenyl-1,2,3,6-tetrahydrophyridine (MPTP), which shows structural similarities with toluene [14]. MPTP is especially potent in depleting central dopamine and inducing lesions in the substantia nigra and induces Parkinson’s disease-like syndrome, much like duodenal [14,15,16] and gastric ulcers [17].

As mentioned, the essential association between dopamine and gastric acid hypersecretion was not immediate. Cysteamine initially went as a model of the duodenal stress ulcer in the patients [1]. Later, duodenal ulcer formation was associated with increased gastric acid output, elevated serum gastrin, and delayed gastric emptying [18,19] and somatostatin, and its inhibition [20].

Then, the dopamine issue [7] and cysteamine-induced duodenal ulcers in rats were prevented by the dopamine agonists bromocriptine, lergotrile, and apomorphine. Contrarily, the dopamine receptor antagonists, haloperidol and pimozide, raised both the severity of duodenal ulcers and the mortality among cysteamine-treated rats [7]. The resolution was thought to be that bromocriptine and lergotrile greatly reduced gastric acid output in the cysteamine-treated rats [7]. The effects of bromocriptine and lergotrile [7] were similar to the effects of metiamide and cimetidine in the cysteamine model system [21]. The particular central dopamine was immediately envisaged with the evidence that large doses of propionitrile or cysteamine in these experiments resulted in a biphasic behavioral response, i.e., stereotypy (e.g., sniffing, licking, biting) followed by akinesia and rigidity [7]. Subsequently, in rats, a single dose of alpha-methyl-p-tyrosine is paired with time-dependent depletion of brain noradrenaline and dopamine levels, which were found to accelerate the development and aggravate the intensity of duodenal ulcers caused by cysteamine while only intracerebroventricular injection of dopamine, but not that of noradrenaline, also inhibited the development of duodenal ulcers [22].

Thus, taking these findings as an initial background, this review focused on the dopamine in the stomach, therapy significance, and the disagreements considering the efficacy of dopamine agonists and efficacy of dopamine antagonists, possibilities still unrealized undertaking that could be, however, realized in future therapy.

## 2. Dopamine Stomach Story before Selye and Szabo Duodenal Ulcer Studies

For the dopamine stomach/duodenum story description, based on Selye and Szabo’s duodenal ulcer studies [1,7], we would emphasize the antecedent evidence [23,24,25]. Determination of the dopamine in enterochromaffin-like cells of the gastric mucosa and gastric DOPA decarboxylase physiological activity was paired with Hakanson’s studies [23]. The presentation of dopamine in human gastric juice was paired with studies of Häggendal and Christensen [24,25]. Dopamine receptors in the stomach went much later [11,26,27].

On the other hand, we should mention that the amphetamine-preventing effect demonstrated much earlier in the reserpine gastric ulcer [28], was considered at that time as a 5-HT-related model [28]. With this consideration [28], the dopamine story as a possible therapy approach in the stomach starts with the preventive effect of fusaric acid, and 5-dimethyldithiocarbamylpicolinic acid, dopamine beta-hydroxylase inhibitors [29,30].

These were subsequent to the evidence that the release of endogenous monoamines by reserpine and tetrabenazine aggravated the gastric ulcer induced by water-immersion stress [31].

Thereby, there is further evidence that intravenous administration of small doses of dopamine (3 µg/kg body weight/min) or L-dopa significantly lowered the development of mucosa erosions [32,33].

This initial evidence is, however, disputed by the notation of the dopamine agonists failure, or effectiveness of the dopamine antagonists or drugs inhibiting catecholamine synthesis [34,35,36].

## 3. Dopamine Stomach Story after Szabo Duodenal Ulcer Studies, 1980–1990

Of note, Szabo’s duodenum study [7] stimulates dopamine gastric ulcer studies, providing strong evidence in favor of the dopamine agonists beneficial effects, apomorphine, d-amphetamine, methylphenidate, and threo-dl-p-hydroxymethylphenidate [37,38], and monooxydase inhibitor L-deprenyl [39] more than for the bromocriptine [40].

Studies of stomach ulcerations in pylorus-ligated rats revealed that a single dose of the dopamine agonists L-dopa, bromocriptine, or apomorphine produced a protective effect [41]. A single dose of the dopamine antagonists haloperidol, sulpiride, or domperidone potentiated the ulcerogenic effect by extending the length of stomach ulcerations [41]. Moreover, an innate ulcerogenic potential is paired with dopamine antagonists. Given alone, without any other ulcerogenic procedure, a single intraperitoneal dose of the dopamine antagonists haloperidol, metoclopramide, or domperidone provoked gastric lesions in all rats within 24 h [42]. The additional focus was on the haloperidol dose-dependence and gastric lesions as early as 90 min after its application and lesions counteraction by simultaneous applications of the dopamine agonists, bromocriptine, or L-dopa [42]. These beneficial effects of the dopamine agonists, as well as the ulcerogenic effects of the dopamine antagonists, were subsequently extended to the mice [43] and to the indomethacin-induced stomach much like small intestine lesions in rats [44]. The extension also included acute pancreatitis attenuation [45].

Further studies were centrally (microinjections) oriented studies [46,47,48,49,50,51,52,53]. Illustratively, microinjections of dopamine or its agonist apomorphine into the central amygdala attenuated cold restraint-induced gastric ulcer formation in rats [46,47]. Contrarily, dopamine antagonists, haloperidol, and metoclopramide reversed the stress ulcer attenuating effect of dopamine [46,47]. Similarly, acute treatments with haloperidol, clozapine, and metoclopramide as well as clozapine chronic treatment significantly facilitated cold-restraint-induced gastric ulcer formation in rats. In addition, haloperidol and clozapine also produced gastric mucosal erosions in non-stressed rats [48]. Along with this was a repeated demonstration of the preventive effect of the dopamine itself, given as an infusion [54] and a dopamine derivative anti-ulcer activity by improving mucosal hemodynamics in the hemorrhagic shock-reperfusion rat model [55].

Together, these studies provided quite consistent evidence that the dopamine antagonists by themselves can induce gastric ulcers and that the dopamine agonists can block the ulcerogenic effect of the dopamine antagonists [42,43,48]. Thus, these studies [42,43,48] fully support Szabo’s original dopamine beneficial concept [7], and counteract most of the pitfalls coming from the opposite view [56] providing the original focus on the duodenal ulcers but not on gastric ulcers. Summarizing the contention achieved at that time, it became clear that dopamine-mimetic agents, which may have different dopamine targets and actions, all possess a strong anti-ulcer beneficial action. They may interact peripherally, and even more centrally [29,30,31,32,37,38,39,40,41,42,43,44,45,46,47,48,49,50,51,52,53,54,55] providing the essential role of the central amygdale nucleus [46,47,48,49,50,51,52,53]. There are interactions with many other systems, i.e., neurotensin [38,47,57], thyrotropin-releasing hormone (TRH) [50], encephalin [52], and prostaglandins [44,53].

Of note, this dopamine issue was covered by several reviews (gastric, pancreatic, and duodenal secretion; gastrointestinal motility; and gastric and intestinal submucosal blood flow regulation in addition to experimental duodenal and gastric ulceration) [12,58,59] providing dopamine as an important enteric neuromodulator and a key element of the “brain-gut axis” and representing a potentially important target for pharmacotherapeutic exploitation [58].

However, some studies still maintained the opposite, the beneficial effect of dopamine antagonists [60,61,62,63,64,65]. This was in a particular case with sulpiride [60,61,62,63,64,65].

## 4. Dopamine Stomach Story after 1990

In the dopamine story, practical solutions appeared in the clinical demonstration. Bromocriptine and amantadine increased initial healing in duodenal ulcer patients with a low relapse rate [66,67,68]. Moreover, there was a favorable comparison of the effects of the dopamine-like drugs bromocriptine and amantadine with the H2 blockers cimetidine and famotidine on healing and relapse rates in a large series of duodenal ulcer patients [69]. There was a significantly lower number of relapsing patients in the group treated with the dopamine-like agents (particularly relative to cimetidine). A simple daily regimen (dose similar to that used in the treatment of less severe disturbances such as ablactation, polycystic ovarian disease, and premenstrual syndrome) showed both the incidence of side effects and the dropout rate not different from those obtained in patients treated with the H2 blockers [69]. 

Naturally, even without mentioning these clinical data [69], the achieved dopamine contention forced further elaboration of the functionally important brain-gut dopamine axis [70,71] emphasizing the significance of the existence of dopamine receptors in human gastric mucosa, likely a D1 subtype [72,73]. Of note, duodenal ulcer patients exhibit proliferation (or up-regulation) of dopamine receptors in the duodenal mucosa [73]. Likewise, the particular importance of the brain dopamine areas, mesolimbic, nigrostriatal, and mesocortical, was based on the counteracting potential against stress gastric ulcers and duodenal lesions of central microinjection of selective dopamine D1 agonists (SKF38393, SKF75670C) and ulcerogenic potential of a D1 antagonist [SCH23390] as mesolimbic nigrostriatal, and none for mesocortical dopamine tracts [74]. Of note, these points were related also to anxiety [75] and aging [76].

On the other hand, it may be that these clinical data [69] encouraged the revealing of the particular significance of the immune cells in the lamina propria [77]. These immune cells were the only cells that showed detectable messenger RNAs for histamine, muscarinic, gastrin, and dopamine receptors by in situ hybridization histochemistry [77]. None of the epithelial cells expressed any of these messenger RNAs [77]. Unfortunately, the evidence of cells of the immune system in the gut and not parietal cells as the targets of antiulcer drugs [77], and the clinical evidence of dopamine agents [69], did not receive general acceptance.

Further studies attempt to specify the dopamine evidence for brain regulation of the gastroduodenal function and pathological responses [78,79,80]. D1 agonists, such as SKF38393 and SKF75670C, reduce experimental gastric mucosal injury and secretion [78]. Antagonists of these receptors, including SCH23390, worsen experimental gastroduodenal lesions and augment secretion [78]. Also, dopamine D3 receptors by 7-hydroxy-N, N-di-n-propyl-2-aminotetralin (7-OHDPAT) are also associated with antisecretory and gastroprotective effects [78]. A similar D1 emphasis was found with SKF38393 in other studies emphasizing the role of both central and peripheral receptors [81,82].

More to the D1 receptors, the emphasis was on the D2 agonist bromocriptine (2.5 or 5.0 mg/kg), which attenuated gastric stress ulcers. Pretreatment of rats with the dopamine depletor alpha-methyl-para-tyrosine or the D1-antagonist SCH23390 clearly neutralized the stress ulcer-attenuating effects of bromocriptine [79]. In this, the next studies [83] may be particularly indicative. Pretreatment with dopamine agonists (bromocriptine, L-dopa, apomorphine) and a histamine H2 receptor antagonist (cimetidine) greatly reduced the hemorrhagic gastric lesions induced by 15 min pylorus ligation in rats [83]. Dopamine antagonists (haloperidol, sulpiride, domperidone) significantly aggravated these lesions. The illustrative novelty is the protective brain-mediated interaction of cimetidine and dopamine systems based on the evidence that cimetidine markedly diminished the ulcerogenic effect of haloperidol but not that of domperidone [83]. Bromocriptine, a dopamine receptor agonist, was emphasized also as an alpha-2 agonist, and its prevention of indomethacin-induced gastric ulcer was inhibited by L-nitro-arginine methyl ester (L-NAME), a nitric oxide synthase (NOS) inhibitor. Thus, increasing levels of NO by bromocriptine may be involved in protective effects on gastric mucosa [84]. Less and shorter-lasting stress-induced increases of corticosterone can explain recovery from a gastric ulcer by apomorphine [85]. Finally, modulation of dopaminergic levels by neurons located within the substantia nigra may play an important role in the development of gastric erosions [86]. However, dopamine could not rescue acetic acid-induced gastric ulcer [87].

Contrarily, the evidence for the dopamine antagonists appears to be in complete contrast with the dopamine agonists evidence (from this period (1969–1990), as well as from the previous periods [12,28,29,30,31,32,33,37,38,39,40,41,42,43,44,45,46,47,48,49,50,51,52,53,54,55,56,57,58,59]). The opposing dopamine antagonists’ evidence appears with the demonstration that a dopamine D4 receptor blockade by clozapine and activation has antisecretory and gastroprotective effects [78,88]. Likewise, a similar claim appeared for sulpiride [89,90] providing that sulpiride, being a dopamine D2 antagonist, behaves much like dopamine D1 agonist fenoldapam [91]. Note that, previously sulpiride (10 or 50 mg/kg) had dose-related effects. The lower dose aggravated, whereas the higher dose attenuated stress ulcerogenesis [80]. Moreover, in rats subjected to activity-stress daily treatment with centrally acting dopamine antagonists, SCH23390 ([R]-(+)-8-chloro-2,3,4,5-tetrahydro-3-methyl-5-phenyl-1H-3-benzazepin-7-ol) (0.1–10 mg/kg), haloperidol (0.1–10 mg/kg), sulpiride (32–320 mg/kg), clozapine (1–100 mg/kg) and metoclopramide (1–100 mg/kg) suppressed the gastric lesion formation [92]. In addition to sulpiride, beneficial effects were suggested also for atypical antipsychotics aripiprazole and risperidone, in distinctive models, indomethacin-, cold and restraint-stress-, pylorus ligation-, stress-immobilization and chronic unpredictable stress-induced gastric ulcers [93,94,95,96]. It was recently shown that zinc salt enhances the gastroprotective activity of risperidone in indomethacin-induced gastric ulcers [93].

## 5. Peptides

Meanwhile, several peptides (i.e., amylin [97], cholecystokinin [98,99], leptin [100], and stable gastric pentadecapeptide BPC 157 [17,101,102,103,104,105,106,107]) were included in the dopamine gastric ulcer story. Illustratively, peripheral administration of amylin (40 µg/kg) may exert a specific gastroprotective effect in the reserpine-induced gastric lesions in the rat (since not effective in other gastric lesions models) [97]. Furthermore, in reserpine-gastric ulcers, its anti-ulcer activity is decreased by pretreatment sulpiride (0.1 mg/kg s.c.) or domperidone, dopamine D2 antagonists. Likewise, SCH 23390, a D1 antagonist, at the maximal dose used, also significantly decreased the gastroprotective activity of the peptide [97]. On the other hand, it may be that the stable pentadecapeptide BPC 157, considering the available evidence [17,101,102,103,104,105,106,107], may approach Szabo’s gastroduodenal ulcer-Parkinson’s disease-schizophrenia circle [7,13]. This may be with its particular modulating effect on the dopamine system [101,102,103,104,105,106,107] (note that BPC 157 competes with the haloperidol-induced catalepsy and gastric ulcer in a non-competitive way [105,107]), and with a brain-gut axis as a mediator [107]. Note that the haloperidol-induced gastric ulcer was specially characterized as dopamine-specific, providing the attenuation by pentadecapeptide BPC 157, omeprazole, bromocriptine, but not atropine, lansoprazole, pantoprazole, ranitidine, cimetidine and misoprostol in mice [104]. Noteworthy, to contribute as a class to the ulcerogenic effect, dopamine antagonists, neuroleptics, and anti-emetics, decreased pressure in the lower esophageal and pyloric sphincters, an effect counteracted by BPC 157 application [108].

## 6. Harmonizing the Dopamine Agonists/Antagonists Issue into the General Therapy Concept

Thus, to summarize the dopamine agonists/antagonists issue into the general therapy concept, we should make additional notes. The viewpoint of gastroprotection should acknowledge the large range of the agents that promote various dopamine functions, and their beneficial effects in both gastric and duodenal ulcers collected during a long period, reviewed in the present study [11,12,28,29,30,31,32,33,37,38,39,40,41,42,43,44,45,46,47,48,49,50,51,52,53,54,55,56,57,58,59,66,67,68,69,70,71,72,73,74,75,76,77,78,79,80,81,82,83,84,85,86,87]. These would strongly support the application of the dopamine agonists as the suited agents in the therapy, and also, Szabo’s original proposal that Parkinson’s disease patients have an increased incidence of ulcers while schizophrenia patients have a decreased incidence of ulcers [7,13]. In support, that point was largely confirmed as well [109]. 

Contrarily, opposing the described dopamine agonists evidence [1,7,11,12,28,29,30,31,32,33,37,38,39,40,41,42,43,44,45,46,47,48,49,50,51,52,53,54,55,56,57,58,59,66,67,68,69,70,71,72,73,74,75,76,77,78,79,80,81,82,83,84,85,86,87], the problematic issue may be the comparable beneficial effect of the dopamine antagonists [34,35,36,60,61,62,63,64,65,78,88,89,90,91,92,93,94,95,96]. Although probably with fewer studies [34,35,36,60,61,62,63,64,65,78,88,89,90,91,92,93,94,95,96], the dopamine antagonists evidence is verified in various animal models and also in clinics [63], and their beneficial effects in both gastric and duodenal ulcers were also accumulated during this long period [34,35,36,60,61,62,63,64,65,78,88,89,90,91,92,93,94,95,96].

A possible outcome of how to harmonize dopamine agonists/dopamine antagonists issue could be Szabo’s original caveats for the dopamine agonists [7]. But, it seems to us, that these caveats are not suited to explain completely the dopamine antagonists issue [34,35,36,60,61,62,63,64,65,78,88,89,90,91,92,93,94,95,96]. Namely, the prolonged treatment (clearly evident anti-ulcerogenic effect of bromocriptine and lergotrile) opposes the single dose with hardly a demonstrable effect [7]. Thereby, there is a supposed switch from the aggravation (one or two large doses of dopamine agonists may aggravate the symptoms and signs of duodenal ulcer [7]) to the beneficial effect (excessive stimulation of dopamine receptors may block the receptor, or transform it to antagonist receptor [7]). On the other hand, among a huge number of studies favoring dopamine agonists’ beneficial effects, there are many studies showing that a single dose of the dopamine agonists exerts a beneficial effect promptly [11,12,28,29,30,31,32,33,37,38,39,40,41,42,43,44,45,46,47,48,49,50,51,52,53,54,55,56,57,58,59,66,67,68,69,70,71,72,73,74,75,76,77,78,79,80,81,82,83,84,85,86,87]. Thus, Szabo’s assumption [7] appeared to be not correct. 

Instead, in practice, we have to acknowledge the more complex evidence that this means two pharmacologically distinct mechanisms with opposite effects on the same (dopamine) signaling pathway produced the same physiological response (gastrointestinal tract protection).

As we mentioned before, considering the stress and cytoprotection concepts for the cysteamine development and application [1,10], we suggest that these different agents can also participate, possibly via the general mechanism of the stress and cytoprotection, due to the stress and cytoprotection effect on the cysteamine, and dopamine system balance. As indicated before, cysteamine, unlike its high-dose range duodenal ulcerogen, given in lower doses, has a positive effect on the gastric mucosa challenged with strong alcohol (attributed to its sulfhydryl capability), known as cytoprotection [10]. Without a stomach, in gastrectomized rats, the cysteamine damaging effect (duodenal lesions, defined as dopamine system blockade [7,13]) was convincingly defined as an essential gastric acid-independent injury, analogous to ethanol gastric lesions, prototypic in cytoprotection studies [110]. BPC 157, ranitidine, omeprazole, atropine, and bromocriptine, prototypic dopamine agonist, counteracted the cysteamine duodenal lesions in the gastrectomized rats, and therefore, act without gastric acid secretion and are cytoprotective [110]. Thus, it may be that cytoprotection, known as the prime prostaglandins’ effect, can be also perceived in dopamine terms [110]. Furthermore, the original cytoprotection terms are extended to the adaptive cytoprotection terms, Robert’s cytoprotection [8] to an additional concept, adaptive cytoprotection [9] (i.e., the initial application of the small irritant (i.e., 15% alcohol) and its mild ulcerogenic effect protect against subsequent strong irritants (and severe ulcerogenic effects)) [9]. Consequently, it may be that the cysteamine, given in lower doses [10], acts also as a dopamine antagonist [7,13], and its positive effect on the gastric mucosa, can be a small irritant effect. That small irritant with small lesions protects against strong irritants’ major ulcerogenic effects, known in adaptive cytoprotection studies [9]. Thereby, it may be that dopamine antagonists, having their own ulcerogenic effect, may act as “small irritants”. Likely, they can exert their beneficial effect against subsequent strong irritants (i.e., absolute ethanol intragastric application). Likewise, there is an overlap between Robert’s and Selye’s terms (mild stress (or) protects against severe stress (or) (Selye’s general adaptive syndrome [111]); small irritant protects against strong irritant (Robert’s adaptive cytoprotection [9])), it may be, considering the essential involvement of dopamine in the stress [112,113], that dopamine antagonists may also act as a “mild stressor”. Thereby, considering the dopamine antagonists’ beneficial effects noted in the studies [93,94,95,96], the dopamine antagonists as “mild stressors” may exert their beneficial effect against subsequent severe stress that may be induced by subsequent stress procedure (i.e., cold + restraint stress; immobilization stress; chronic unpredictable stress). On the other hand, these have to be under particular stress conditions, providing that otherwise in many studies, dopamine antagonists can facilitate stress procedures-induced gastric lesions [46,47,49,51,52,53,79,80].

## 7. Conclusions

In summary, this dopamine gastric ulcer healing review takes distinctive duodenal key points, the Selye and Szabo description of the cysteamine-induced duodenal ulcer in rats as duodenal stress ulcer in patients [1], Szabo’s cysteamine duodenal ulcer as the dopamine duodenal healing, cysteamine as a dopamine antagonist, dopamine agonists (anti-ulcer effect), and dopamine antagonists (ulcerogenic effect) [7,13]. From these viewpoints, we focused on dopamine and gastric ulcer healing. Firstly, we mentioned antecedent studies on the dopamine presence in the stomach and gastric juice [23,24,25]. Then, we reviewed, in the timeline, therapy significance, and possibilities coming from the anti-ulcer potency of the various dopamine agonists and the ulcerogenic effect of the dopamine antagonists [11,12,28,29,30,31,32,33,37,38,39,40,41,42,43,44,45,46,47,48,49,50,51,52,53,54,55,56,57,58,59,66,67,68,69,70,71,72,73,74,75,76,77,78,79,80,81,82,83,84,85,86,87], which is highly prevailing over the quite persistent beneficial evidence arising from the various dopamine antagonists [34,35,36,60,61,62,63,64,65,78,88,89,90,91,92,93,94,95,96,97].

Meantime, the beneficial effects of several peptides (i.e., amylin [97], cholecystokinin [98,99], leptin [100], and stable gastric pentadecapeptide BPC 157 [17,101,102,103,104,105,106,107] (suggested as the acting mediator of the dopamine brain-gut axis [107])) further extended the dopamine gastric ulcer story.

Finally, we combined the known concepts backing cysteamine development and application to harmonize dopamine agonists/antagonists issue into a general therapy concept. We considered the dopamine significance in stress [112,113] (cysteamine as a prototype of the duodenal stress ulcer [7]) and cytoprotection (cysteamine in small dose as a prototype of the cytoprotective agents [10]; cysteamine duodenal ulcer in gastrectomized rats lesions [110]). Thereby, in special circumstances (since dopamine antagonists otherwise facilitated stress-induced gastric ulcer [46,47,49,51,52,53,79,80]), the own ulcerogenic effect of dopamine antagonists may act as a “mild stress (or)” or “small irritant” to counteract subsequent strong alcohol- or stress procedure-induced severe lesions in this particular stomach target. Although not previously mentioned, this speculation may at least partly explain the observed beneficial effect of dopamine antagonists’ application [93,94,95,96,97]. This may be a particular case, since dopamine antagonists act as a class and have shared adverse effects (i.e., prolonged QT interval [101,114]). Similarly, caution can be taken with the dopamine agonists’ possible adverse effects [112,113]. On the other hand, cysteamine, when given as an enema, may induce ulcerative colitis lesions [115,116,117]. These may likely extend the effectiveness of the gastroduodenal anti-ulcer agents [115,116,117], the dopamine agents, in particular, as suggested [112], on the lesions of the lower gastrointestinal tract as well [112]. Finally, the development of the new agents [112], or, possibly, mentioned peptides application (i.e., amylin [97], cholecystokinin [98,99] leptin [101] and stable gastric pentadecapeptide BPC 157 [102,103,104,105,106,107,108,110,113,114,115,116,117] likely acting mediator of the dopamine brain-gut axis [107]) may likely approach the original gastroduodenal ulcer-Parkinson’s disease-schizophrenia circle [7,13,109].

**Figure 1:**
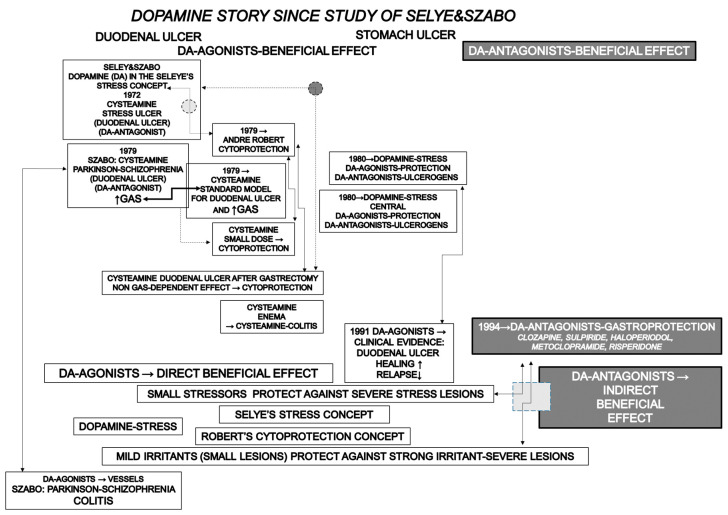
This review attempts to emphasize the significance of dopamine agonists in peptic ulcer disease and to reconcile the gastric acid hypersecretion vs. cytoprotection/stress (non-gastric related), beneficial effects of dopamine agonists vs. the beneficial effects of dopamine antagonists. Briefly, the contemporary dopamine (DA) story starts with the evidenced dopamine antagonist cysteamine duodenal lesions within the frame of the Selye’s stress concept [5], as a stress lesion prototype (see ref. [1]), revised a few years later to the gastric acid secretion (GAS) (hypersecretion)-induced duodenal ulcer, which became standard duodenal ulcer model for anti-ulcer agents screening procedure [7]. In this, with the cysteamine dopamine antagonist, Parkinson-schizophrenia-ulcer dopamine relation is suggested to provide a general background for dopamine agonists application in the ulcer disease [7]. The parallel breakthrough was Robert’s cytoprotection concept, cytoprotection, and the non-gastric acid secretion-related effect [8]. Subsequently, with adaptive cytoprotection, Robert [9] adopted Selye’s quotations [5,111] in his stress concept (Robert’s small irritants-induced small lesions that protect against strong irritant-induced severe gastric lesions fairly correspond to Selye’s small stress (or) protection against severe stress) [9]. To the dopamine significance in the adaptive cytoprotection goes the evidence that the small dose of the cysteamine (i.e., small irritant) was gastroprotective [10]. Meanwhile, in the counteracting stomach lesions, dopamine evidence was thought to be non-gastric acid dependent, and dopamine agonists’ beneficial effect in the stomach lesions healing follows dopamine significance in the stress. This point was further supported by the establishing central significance of dopamine agonists’ beneficial effects [37,38,39,40,41,42,43,44,45,46,47,48,49,50,51,52,53]. Final evidence was the clinical evidence that dopamine agonists (bromocriptine, amantadine) increase duodenal ulcer healing and prevent relapse over H2 blockers (famotidine) [69]. Definitive departure from gastric acid (hyper)secretion with cysteamine (dopamine antagonist)-duodenal ulcer back toward the original Selye’s and Szabo’s non-gastric acid-related effect [1] was the evidence that cysteamine may induce duodenal lesions in the gastrectomized rats, a clue for the cytoprotective background [110]. Subsequently, cysteamine enemas produced severe colitis lesions [115,116,117]. Meanwhile, there is growing evidence for dopamine antagonists’ beneficial effects [78,88,93,94,95,96]. Finally, to reconcile the dopamine agonists/antagonists issue into the general therapy concept, we considered the dopamine significance in the stress [112,113] (cysteamine as prototype of the duodenal stress ulcer [7]) and cytoprotection (cysteamine in small dose as prototype of the cytoprotective agents [10]; cysteamine duodenal ulcer in gastrectomized rats lesions [110]) concepts for the cysteamine development and application. Thereby, in special circumstances (since dopamine antagonists otherwise facilitated stress-induced gastric ulcer [46,47,49,51,52,53,79,80]), the own ulcerogenic effect of dopamine antagonists may act as a “mild stress (or)” or “small irritant” to counteract subsequent strong alcohol- or stress procedure-induced severe lesions in this particular tissue. Although not previously mentioned, this speculation may at least partly explain the observed beneficial effect of dopamine antagonists’ application [93,94,95,96]. This summary did not include the beneficial effects of the several peptides (i.e., amylin, cholecystokinin, leptin, and stable gastric pentadecapeptide BPC 157, suggested as acting mediator of the dopamine brain-gut axis) which were included in the dopamine gastric ulcer story [97,98,99,100,101,102,103,104,105,106,107,108].

Concluding, this challenging topic, the “dopamine story” as perceived by the authors, is summarized in Figure 1.

Therefore, for resolution, we should go outside of the original topic of this review, providing dopamine in the stomach as the main issue. Instead of a conclusion for dopamine agonist/dopamine antagonist issue, we should, as a new improvement in further therapy, conclude with dopamine significance in inflammatory bowel disease (patients with Parkinson’s disease have elevated colonic levels of pro-inflammatory cytokines and increased intestinal permeability, which are hallmark signs of inflammatory bowel disease pathogenesis) [118,119]. Illustratively, the novel ulcerative colitis evidence provides that D2 receptor agonists decreased the severity of ulcerative colitis in two animal models (for review see, i.e., [118,119]). The attenuation of enhanced vascular permeability and prevention of excessive vascular leakage may likely indicate further ways of the dopamine agonists’ activity application [118,119].

## Data Availability

Data sharing is not applicable.
